# Quantitative identification of ventral/dorsal nerves through intraoperative neurophysiological monitoring by supervised machine learning

**DOI:** 10.3389/fped.2023.1118924

**Published:** 2023-05-18

**Authors:** Wenbin Jiang, Qijia Zhan, Junlu Wang, Min Wei, Sen Li, Rong Mei, Bo Xiao

**Affiliations:** ^1^ Department of Neurosurgery, Shanghai Children’s Hospital, School of Medicine, Shanghai Jiao Tong University, Shanghai, China; ^2^ Department of Neurology, Shanghai Children's Hospital, School of Medicine, Shanghai Jiao Tong University, Shanghai, China

**Keywords:** spastic cerebral palsy, selective dorsal rhizotomy, neurophysiology, intraoperative monitoring, machine learning

## Abstract

**Objective:**

This study aimed to investigate the electro-neurophysiological characteristics of the ventral and dorsal nerves at the L2 segment in a quantitative manner.

**Methods:**

Medical records of consecutive patients who underwent single-level approach selective dorsal rhizotomy (SDR) from June 2019 to January 2022 were retrospectively reviewed. Intraoperative electro-neurophysiological data were analyzed.

**Results:**

A total of 74 males and 27 females were included in the current study with a mean age of 6.2 years old. Quadriceps and adductors were two main muscle groups innervated by L2 nerve roots in both ventral and dorsal nerve roots. Dorsal roots have a higher threshold than that of the ventral ones, and muscles that first reached 200 µV innervated by dorsal roots have longer latency and smaller compound muscle action potential (CMAP) than those of the ventral ones. Supervised machine learning can efficiently distinguish ventral/dorsal roots using threshold + latency or threshold + CMAP as predictors.

**Conclusion:**

Electro-neurophysiological parameters could be used to efficiently differentiate ventral/dorsal fibers during SDR.

## Introduction

Selective dorsal rhizotomy (SDR), an effective neurosurgical measure to decrease spasticity mainly in patients suffering from spastic cerebral palsy, is generally adopted to improve their motor function and has been practiced for decades (1, 2). The surgical team is required to differentiate dorsal spinal nerve roots from those ventral ones in the cauda equina as the first step in the selection during such a procedure (3). When SDR is performed under a multi-level (4, 5) or a less-traumatic keyhole interlaminar approach (6, 7), such differentiation could be achieved by the natural anatomy of those nerve roots at the nerve exit from the dura sac at a certain level (8). The operators could also differentiate nerve roots by comparing their neurophysiological characteristic differences, such as the electrical threshold to elicit electromyography (EMG) responses in monitored muscles (9), with both lower limbs and anal sphincter included (10). Given the lack of anatomical references for those spinal nerve roots through limited surgical exposure in a single-level laminectomy SDR, surgeons are given no alternative but to completely rely upon the interpretation of evoked EMG responses when stimulating a certain nerve root for decision-making (3, 11, 12).

The electrical threshold of a dorsal spinal nerve root is generally 10 times greater than that of a ventral one to evoke EMG responses in a muscle monitored (3). However, it is observed during daily clinical practice that thresholds of some dorsal roots are lower than 0.30 mA, some even approaching 0.20 mA, similar to those of the ventral ones. In the tertiary center where the author worked, the single-level approach SDR through L2 is applied to patients who have been suffering from spastic cerebral palsy and have met the inclusion criteria of the surgery (13). The nerve exit of L2 at the dura sac needs to be exposed during the procedure. To ensure the stability of the intraoperative monitoring system and the appropriate anesthetic status, the nerve roots are tested (using a restricted stimulation protocol) in an order from ventral to dorsal before the stimulation in the rest of the nerve roots in the cauda equina to accomplish the selection of those dorsal fibers for sectioning.

Such an SDR practice enables the scholars to investigate the electro-neurophysiological characteristics of the ventral and dorsal nerve roots of L2 in a quantitative manner and enriches their understanding of the neurophysiological differences between spinal ventral and dorsal nerves. We present the following article/case in accordance with the STARD reporting checklist.

## Methods and materials

A retrospective analysis was performed in consecutive patients with spastic cerebral palsy who had undergone L2 approach SDR from June 2019 to January 2022 upon approval from the institutional review board. This study was conducted in accordance with the relevant guidelines and the Declaration of Helsinki. It has been approved by the Ethics Review Committee, Children's Hospital of Shanghai, Shanghai Jiao Tong University (Approval No.: 2020R069-E02). All SDR surgeries were performed by BX. The inclusion criteria for SDR were listed in previous studies (13). The muscle tone of the lower extremities in all these patients was evaluated by the multidisciplinary team with Modified Ashworth Scale (MAS) for surgical requirements (14). Subjects for further research were also the characteristics of nerve roots in the L2 segment as the root property could be verified by anatomical location.

### SDR procedure, intraoperative anesthesia, and monitoring criterion

**Figure 1:**
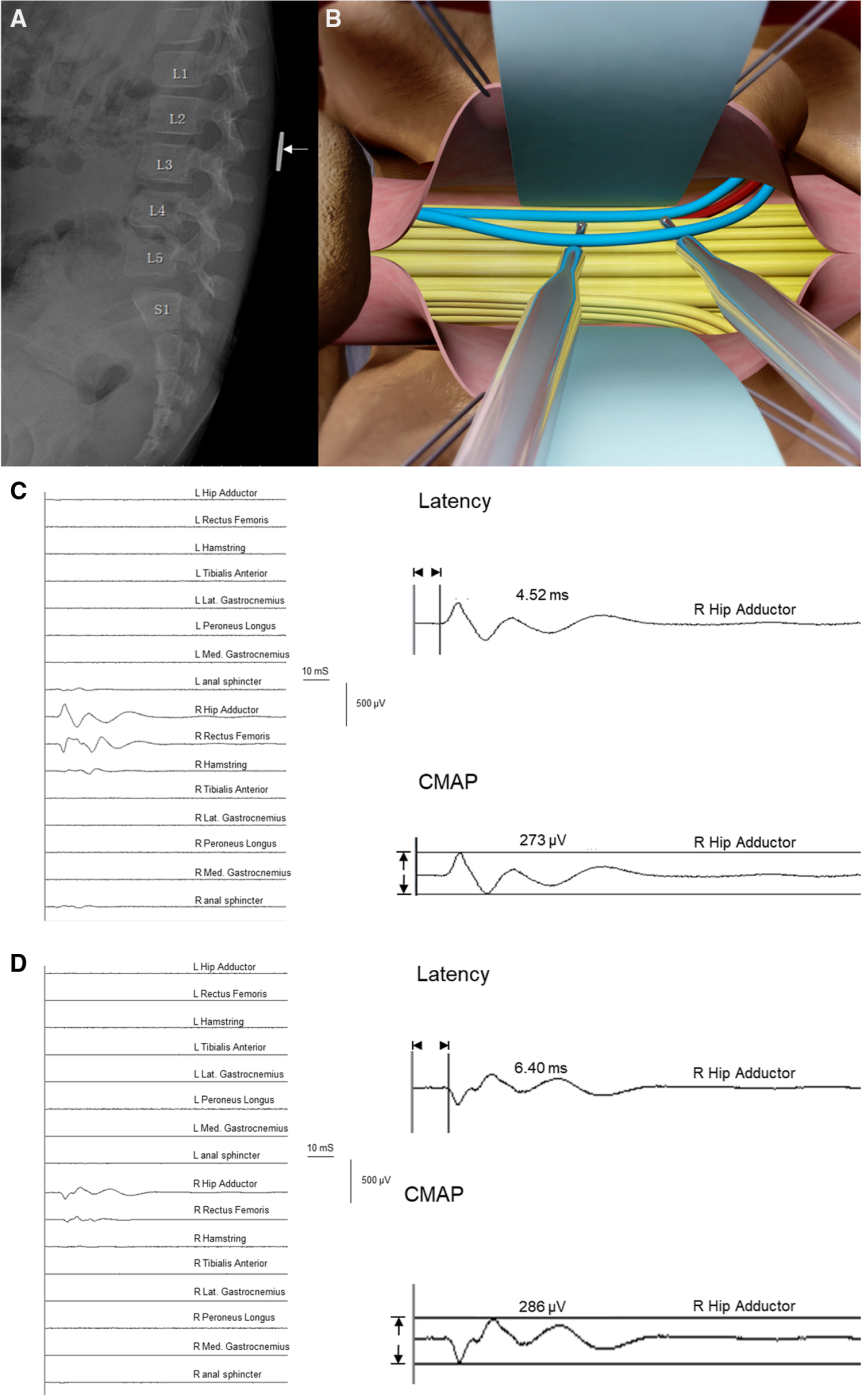
Selective dorsal rhizotomy (SDR) diagram and examples demonstrating electro-neurophysiological characteristics of ventral/dorsal nerve roots. (**A**) X-ray of lumbosacral vertebral for the pre-operational location of L2 with a coin placed on the back of children. (**B**) Illustration of root testing during SDR procedure. Blue: dorsal roots at L2 dual exit. Red: ventral roots at L2 dual exit. Yellow: other roots of the cauda equina shown in the exposure at the level of L2. (**C**) Trigger stimulation of a ventral root at the right L2 dual exit. The threshold was 0.09 mA, the *Muscle-200* of this root was the right hip adductor with a compound muscle action potential (CMAP) value of 273 µV, and the latency of this muscle was 4.52 ms. (**D**) Trigger stimulation of a dorsal root at the right L2 dual exit. The threshold was 2.90 mA, the *Muscle-200* of this root was the right hip adductor with a CMAP value of 286 µV, and the latency of this muscle was 6.40 ms.

On the day before the surgery, we will attach a coin to the back of the patient to locate the L2 spinous process (Figure 1A). A specific illustration of the procedure was previously reported (12). After the patient was transferred to the operating room, routine electrocardiogram monitoring was initiated. Anesthesia induction was achieved by administering intravenous midazolam (1 mg), propofol (2–3 mg/kg), sufentanil (0.2 μg/kg), rocuronium (0.6 mg/kg), and atropine (0.01 mg/kg). Subsequently, tracheal intubation was performed under visual laryngoscope guidance with volume-controlled ventilation, and the ventilation pressure was adjusted to maintain end-tidal carbon dioxide at 35–45 mmHg. The patient was positioned in a prone position after induction of anesthesia. During surgery, anesthesia was maintained with 0.5 minimum alveolar concentration of sevoflurane, 6–8 mg/(kg·h) of propofol, and 0.1–0.3 μg/(kg·min) of remifentanil administered by infusion pump. Additionally, a water blanket was used to maintain the patient's body temperature between 36.0°C and 37.0°C. Muscle relaxants were used for intubation only. Nerve fibers were visualized after laminectomy at L2 and the incision at the dura mater and arachnoid (Figure 1B).

Cascade®'s intraoperative neuromonitoring system was adopted during the SDR procedure for electrical stimulation of nerve fibers and recording of muscles. EMG monitors were placed bilaterally in the muscles of the legs including adductors, quadriceps, hamstrings, tibialis anterior, lateral gastrocnemius, peroneus longus, medial gastrocnemius, and anal sphincter. Single-pulse (trigger) electrical stimulation and train stimulation were applied to identify nerve roots, and dorsal roots meeting the rhizotomy protocol would be cut partially. Electrical stimulation of nerve fibers should start at least 30 min after applying muscle relaxants. “Free-running” mode would be followed to ensure a peak-to-peak EMG amplitude of less than 20 µV before the beginning of every single electrical stimulation in all monitored channels; otherwise, the judgment of EMG appearance would be influenced. Each tested root was lifted in the air by the stimulating probe electrodes with no tension, and the surgical assistant would place Cottonoid® beneath the roots to guarantee the dryness of the surgical area.

Trigger stimulation (waveform: rectangular) started at 0.00 mA, with a duration of 0.2 ms, and the stimulus interval was 1 s. The step width was 0.01 mA when the current intensity was lower than 0.2 mA and ranged from 0.01 mA to 0.10 mA when the intensity reached over 0.2 mA. The maximum stimulus intensity was 4.0 mA. The rhizotomy protocol was previously discussed in a published article (9). According to the protocol, two things need to be considered carefully when stimulating every single nerve root: (1) whether the rootlet stimulated was motor or sensory and (2), if it was sensory rootlet, whether the stimulation activates target muscles (MAS grade 2 or greater) and should be cut.

### Parameters in neurophysiological data

*Threshold-200* (described as threshold) of certain nerve roots was defined as the current intensity that could first evoke 200 µV (±10%) EMG amplitude in one of the monitored muscles according to the criterion stipulated in advance. The corresponding muscle that first reached 200 µV was defined as *Muscle-200* innervated by this root. *Latency-200* and the peak-to-peak compound muscle action potential (*CMAP-200*) of *Muscle-200* were recorded by the monitor system and documented as latency and CMAP (Figures 1C,D).

A special condition should be stated that the intensity would not be elevated for the protection of nerve fibers if no EMG amplitudes reached 200 µV when the current intensity was 4.0 mA. Under such a circumstance, when the electrical stimulus reached 4.0 mA, if no EMG amplitudes reached 200 µV in any monitored channel, then the monitored muscle with the largest CMAP is defined as *Muscle-200*, and the *Threshold-200* would be defined as 4.0 mA. The time between the onset of muscle relaxants and the time of nerve stimulation was also accounted for.

### Supervised machine learning (binary classification)

Supervised machine learning methods were adopted to binarily classify roots into the ventral and dorsal fibers. Nerve roots classification was performed under the MATLAB classification learner application (version 2021a, MathWorks, Natick, MA). “Threshold + CMAP,” “threshold + latency,” “CMAP + latency,” and “threshold + CMAP + latency” were separately used as predictors to classify all roots. Classification performances were tested in the domains of decision tree, discriminant analysis, logistic regression classifier, Naïve Bayes classifier, support vector machine (SVM), *k*-nearest neighbor (kNN) classifier, and neural network (NN) classifier (15), all of which are part of the MATLAB classification learner toolbox (16).

All models were used with the percentage of training of 10-fold cross-validation for the classification, with metrics including accuracy, precision, recall (sensitivity), F1 score, and specificity correspondingly calculated. A detailed definition of these metrics can be found in the paper published by Petrescu (17).

### Statistical analysis

Continuous variables with normal distribution were presented as mean ± SD, and non-normal variables were reported as median (Q1, Q3). Former variables were compared through the *t*-test or the non-parametric Mann–Whitney *U* test, as appropriate. Statistical comparison is carried out *via* the *χ*^2^ test, and continuity correction was applied whenever appropriate for categorical data. Receiver operating characteristic (ROC) curves were generated with different single factors, and the area under the curve (AUC), measurement of sensitivity, and specificity were thus calculated. A value of *p* < 0.05 is considered statistically significant. Collected data were analyzed by SPSS version 24.0 for Windows (SPSS Inc., Chicago, IL, USA).

## Results

In a total of 101 cases, 74 males and 27 females with a mean age of 6.2 years were retrospectively reviewed (Table 1). During SDR in these patients, 585 nerve roots at the L2 segment were tested, among which 381 nerve fibers were dorsal (left 193, right 188) and 204 were ventral (left 97, right 107). The median time from nerve root detection to the last use of muscle relaxant was 152 min, ranging from 36 to 379 min. The median threshold of roots was 0.79 mA (ranging from 0.01 to 4.0 mA). The EMG amplitude of corresponding *Muscle-200* was 223 µV in median (ranging from 20 to 1999 µV), and the median latency was 5.0 ms (ranging from 2.71 to 16.50 ms).

**Table 1:** Demographic details of included cases and characteristics of nerve rootlets at the L2 level of these cases during selective dorsal rhizotomy (SDR).

Characteristics	No
Gender (*n*)	
Boy	74
Girl	27
Age at SDR (mean ± SD) (years old)	6.2 ± 2.3
GMFCS	2.7 ± 0.9
GMFM-66	61.1 ± 12.7
L2 rootlets tested during surgery (*n*)	
Right	295
Left	290
Motor	204
Sensory	381
Time of electrical stimulation of rootlets (from the use of muscle relaxant, min)	152.0 (119.5, 213)
Threshold (mA)	0.79 (0.16, 2.33)
CMAP (µV)	223.0 (193.5, 368.0)
Latency (ms)	5.0 (4.33, 6.43)

**Figure 2:**
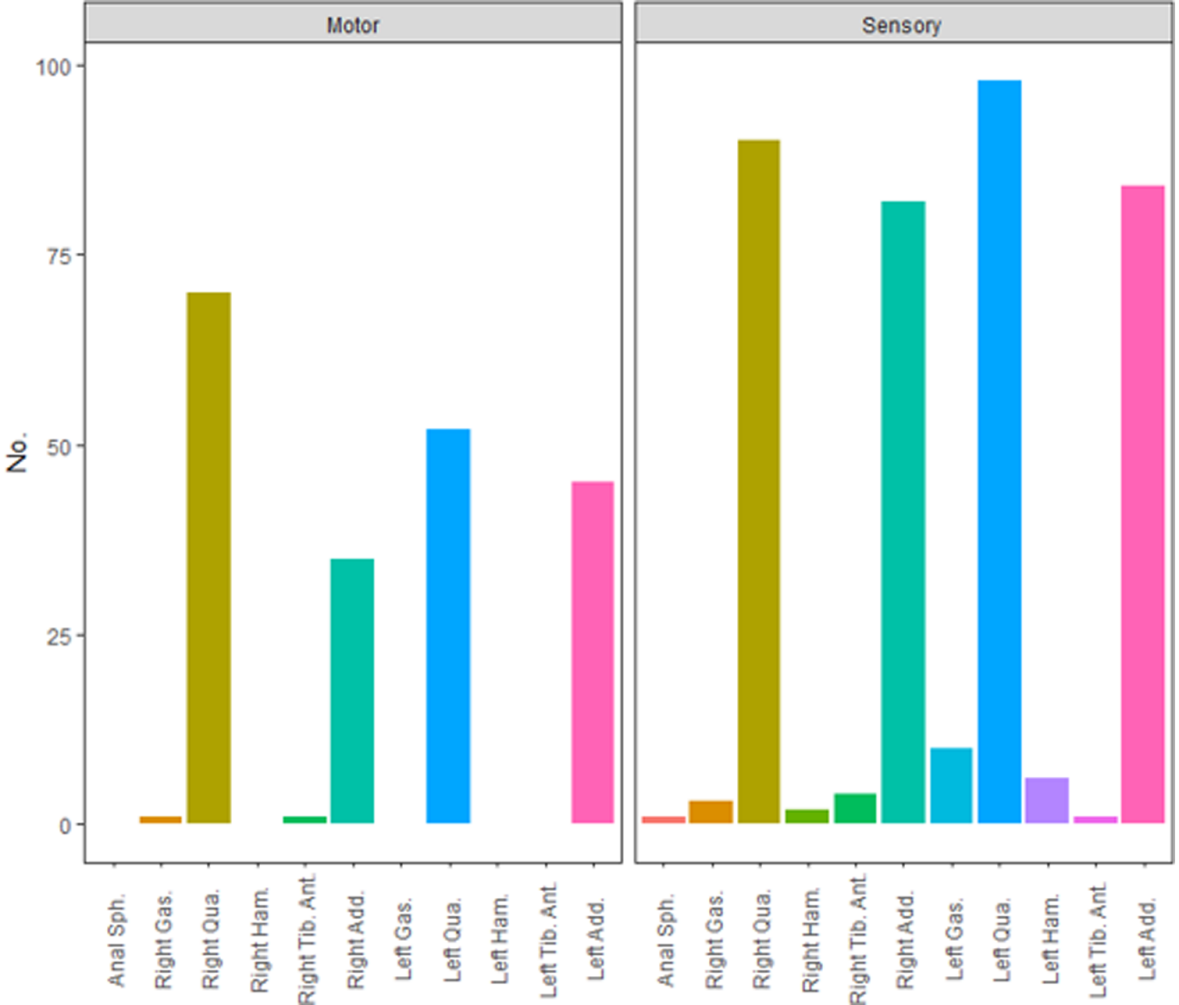
Distribution of *Muscle-200* of ventral and dorsal roots at the L2 level. Anal Sph., anal sphincter; Gas.,gastrocnemius; Qua., quadriceps; Ham., hamstring; Tib. Ant., tibialis anterior; Add., adductor.

The distribution of *Muscle-200* was displayed in Figure 2. Quadriceps and adductors were two main muscle groups innervated by L2 nerve roots in both ventral and dorsal nerve fibers. Among 204 ventral roots, *Muscle-200* was distributed into 122 quadriceps, 80 adductors, one gastrocnemius, and one tibialis anterior. Corresponding *Muscle-200* of dorsal roots was distributed into 188 quadriceps, 166 adductors, eight hamstrings, 13 gastrocnemii, five tibialis anterior, and one anal sphincter. Among 27 *Muscle-200* out of the innervation pattern except for the anal sphincter, 20 were with abnormal muscle tone (MAS grade 2 or greater).

**Figure 3:**
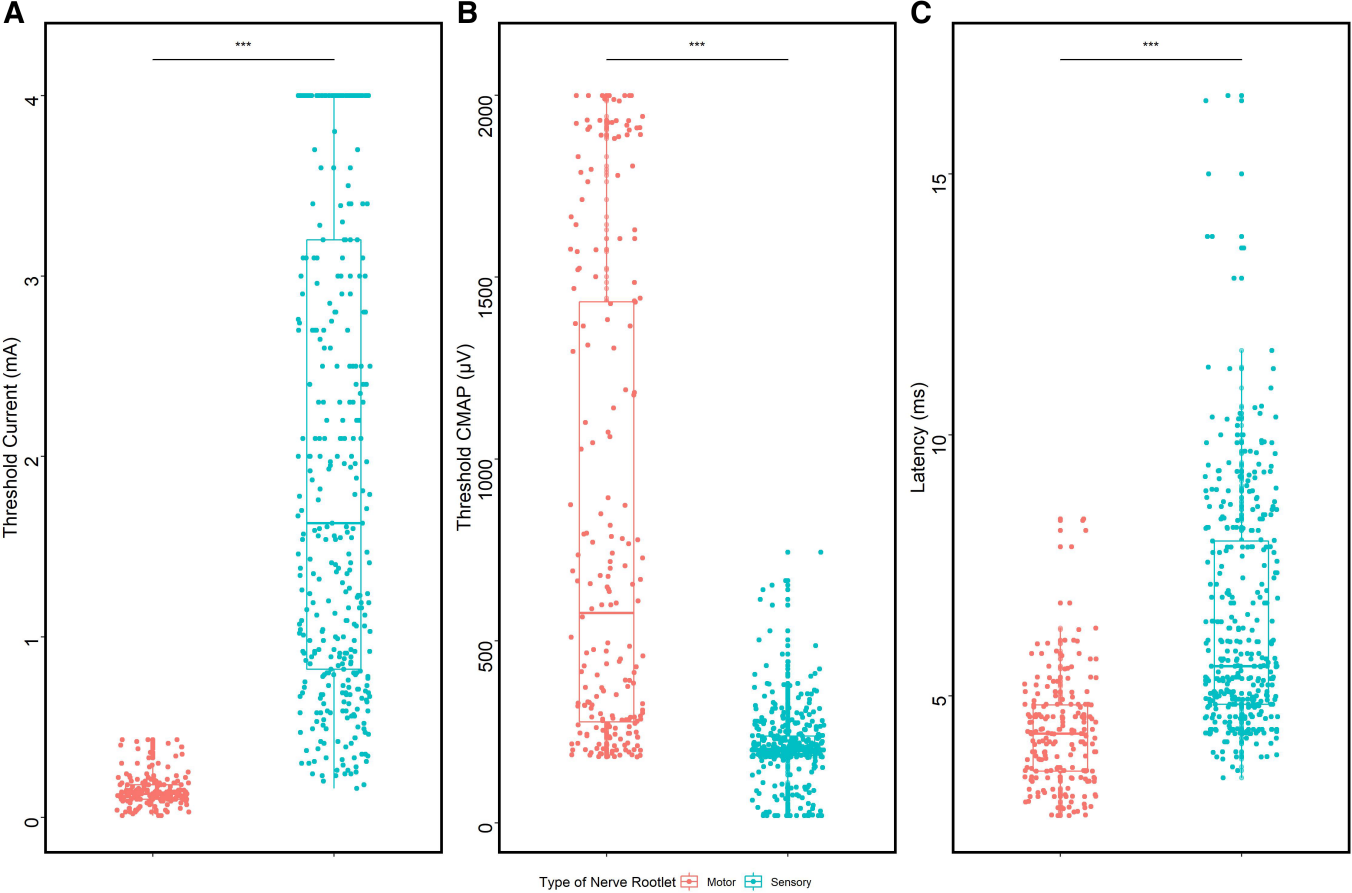
Comparison of ventral and dorsal nerve fibers. (**A**) Threshold comparison of ventral/dorsal roots. (**B**) CMAP comparison of *Muscle-200* evoked by ventral/dorsal roots. (**C**) Latency comparison of *Muscle-200* evoked by ventral/dorsal roots.

Ventral and dorsal fibers exhibited different electrophysiological properties when stimulated with single-pulse current intensity (Figure 3). The median threshold of ventral roots was 0.13 mA (0.10, 0.18), much lower than that of dorsal fibers (median 1.63 mA, Q1: 0.82 mA, Q3: 3.24 mA, *p* < 0.0001). Ventral roots were more easily excitable, in which, in detail, the evoked EMG amplitudes were larger than dorsal roots when stimulated with threshold current intensity, and the comparison in the median was 577.0 µV (274.5 µV, 1,434.0 µV) vs. 201.0 µV (184.5 µV, 238.5 µV), with the statistical analysis result of *p* < 0.0001. The latency of *Muscle-200* was proven to be shorter in ventral roots (4.04 ± 1.44 ms), compared with that in dorsal roots (6.43 ± 2.20 ms, *p* < 0.0001).

**Figure 4:**
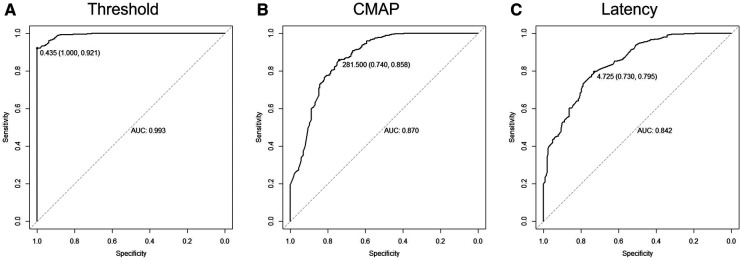
Classifying rootlets with a single electro-neurophysiological factor. (**A**) Receiver operating characteristic (ROC) curve using threshold as a classifier for the identification of ventral/dorsal nerve roots. When the cutoff point was 0.435 mA, the area under the curve was 0.993 (sensitivity, 100%; specificity, 92.1%). (**B**) ROC curve using CMAP as a classifier for the identification of ventral/dorsal nerve roots. When the cutoff point was 281.5 µV, the area under the curve was 0.870 (sensitivity, 74.0%; specificity, 85.8%). (**C**) ROC curve using latency as a classifier for the identification of ventral/dorsal nerve roots. When the cutoff point was 4.73 ms, the area under the curve was 0.842 (sensitivity, 73.0%; specificity, 79.5%).

ROC curves were drawn with three different single electrophysiological factors for binary classification (Figure 4). The largest AUC was obtained by a cutoff point of 0.435 mA (AUC = 0.993, *p* < 0.0001) when the threshold was adopted as the predictor whose accuracy was 94.87%; specificity, 92.1%; and sensitivity, 100% (Figure 4A). CMAP (AUC = 0.870, *p* < 0.0001) and latency (AUC = 0.842, *p* < 0.0001) are also endowed with good performance for the identification of nerve roots.

For the exploration of the method to distinguish dorsal roots from ventral ones efficiently (with the highest accuracy), seven different supervised machine learning models were applied for binary classification (Table 2). The highest accuracy (95.90%), with two factors applied (“threshold + CMAP,” “threshold + latency,” “CMAP + latency”) as predictors, was achieved either by DT, LR, and SVM with “threshold + latency” as a predictor or by kNN with threshold + CMAP as a predictor. With all three factors taken as predictors, the highest accuracy was 95.73% when the machine learning model was DT or LR.

**Table 2:** Highest accuracy calculated by different predictors with different machine learning methods.

Predictors	Model	Accuracy	Precision	Recall	Specificity	F1 score
Threshold + CMAP + latency	DT	0.9573	0.8943	0.9951	0.9370	0.9420
	DA	0.9419	0.9250	0.9069	0.9606	0.9158
	LR	0.9573	0.9324	0.9461	0.9633	0.9392
	NB	0.9521	0.9583	0.9020	0.9790	0.9293
	SVM	0.9556	0.9279	0.9461	0.9606	0.9369
	kNN	0.9470	0.9023	0.9510	0.9449	0.9260
	NN	0.9573	0.9282	0.9510	0.9606	0.9395
Threshold + latency	DT	**0** **.** **9590**	0.8982	0.9951	0.9396	0.9442
	DA	0.9487	0.9183	0.9363	0.9554	0.9272
	LR	**0**.**9590**	0.9327	0.9510	0.9633	0.9417
	NB	0.9521	0.9190	0.9461	0.9554	0.9324
	SVM	**0**.**9590**	0.9167	0.9706	0.9528	0.9429
	kNN	0.9470	0.9139	0.9363	0.9528	0.9249
	NN	0.9556	0.9279	0.9461	0.9606	0.9369
Threshold + CMAP	DT	0.9470	0.9347	0.9118	0.9659	0.9231
	DA	0.9419	0.9250	0.9069	0.9606	0.9158
	LR	0.9504	0.9147	0.9461	0.9528	0.9301
	NB	0.9521	0.9356	0.9265	0.9659	0.9310
	SVM	0.9504	0.9070	0.9559	0.9475	0.9308
	kNN	**0**.**9590**	0.9369	0.9461	0.9659	0.9415
	NN	0.9521	0.9231	0.9412	0.9580	0.9320
Latency + CMAP	DT	0.8496	0.9028	0.6373	0.9633	0.7471
	DA	0.8359	0.9576	0.5539	0.9869	0.7019
	LR	0.8547	0.8839	0.6716	0.9528	0.7632
	NB	0.8547	0.8742	0.6814	0.9475	0.7658
	SVM	0.8513	0.9333	0.6176	0.9764	0.7434
	kNN	0.8564	0.8409	0.7255	0.9265	0.7789
	NN	0.8513	0.8503	0.6961	0.9344	0.7655

DT, decision tree; DA, discriminant analysis; LR; logistic regression; NB, Naïve Bayes; SVM, support vector machine; kNN, *k*-nearest neighbor; NN, neural network.

Bold indicates the highest accuracy achieved by different predictors with different machine learning methods.

**Figure 5:**
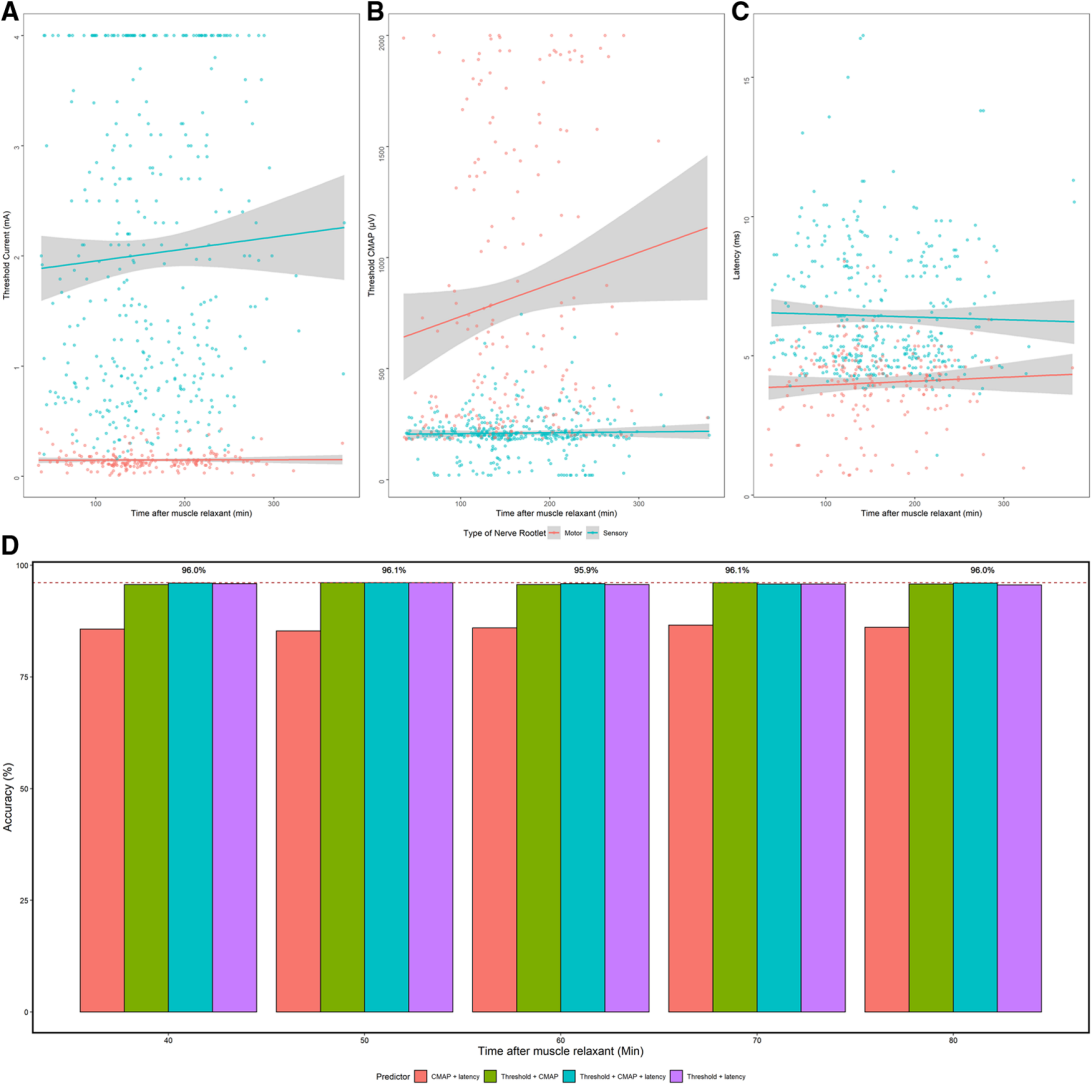
The effect of muscle relaxants on electrophysiological data and on the accuracy of machine learning. (**A–C**) Threshold, CMAP, and latency change over the time after usage of muscle relaxants. Gray bar: linear regression of electrophysiological data of ventral and dorsal nerves. (**D**) The highest accuracy got by supervised machine learning using different predictors in different time intervals after the administration of muscle relaxant. *x*-axis: time after the administration of muscle relaxant when stimulating the nerve roots. *y*-axis: highest accuracy achieved by different methods. Red dash line: highest accuracy (96.1%).

All cases, except for one, adopted muscle relaxants intraoperatively for intubation. As shown in Figures 5A,B, both threshold and CMAP elevated as time passed, but no significant correlation was found neither between threshold and time after muscle relaxant nor between CMAP and time. To explore whether the characteristic change of nerve roots confused the determination of ventral/dorsal fibers, we calculated the highest accuracy using different machine learning methods with different predictors was compared. It showed that accuracy for the identification was 96.0% ± 0.1% if the electrical stimulation start 40 min after the application of the muscle relaxant. The highest accuracy was first achieved at 50 min and 70 min (96.1%) after applying muscle relaxant, and accuracy in other time intervals remained high as well (Figure 5D).

## Discussion

In clinical practice, myotome/dermatome is used to describe the correlation between muscles and spinal nerves in different segments (18). Given its guidance for the operation from an anatomical aspect, especially in the epoch with the absence of intraoperative monitoring, myotome/dermatome is vital for the implementation of SDR. The quadriceps femoris and hip adductor are parts of the L2 myotome, which perfectly coincides with the findings here (19). The electrical stimulation of L2 ventral roots generated max muscle contraction mainly in the quadriceps (62.6%) and adductors (36.1%), but exceptions still existed when one ventral root elicited max EMG amplitude in gastrocnemius and another in tibialis anterior. A dermatome depicts an area of skin innervated by a single dorsal spinal nerve. The dermatome of the L2 segment covers the front and inner side of the thigh, with quadriceps and hip adductors included (20). Based on the retrospective data acquired, 49.3% and 43.6% of the *Muscle-200* were quadriceps and adductors, respectively. The other 7.1% included the anal sphincter, gastrocnemius, hamstrings, and tibialis anterior.

Significant differences were found in electrophysiological characteristics between ventral and dorsal spinal nerves, which made it possible for surgeons to identify different nerve fibers during single-level SDR solely on interpreting the neurophysiological monitoring data. Data acquired demonstrated that the threshold of ventral nerves was almost 15 times higher than that of dorsal ones. Meanwhile, the median EMG amplitude of *Muscle-200* evoked by ventral nerve fibers at threshold stimulation was two times the value generated by dorsal ones. These statistical differences were consistent with the previous research (3). Except for the threshold and CMAP of *Muscle-200* whose latency elicited by ventral nerve roots was 2.4 ms shorter than that innervated by dorsal roots, which was thought to result from synapse delay for the stimulation transmission (21). When the ventral nerves were stimulated, the electrical stimulation would induce muscle activity in the effector with less synapse, and the stimulation given to the dorsal nerve fibers would be first transduced to the interneuron in the spinal cord for further transmission to the effector.

One of the main purposes of this research is to analyze the difference between ventral and dorsal nerve roots, seeking the best way of identifying them. If only one indicator was used to distinguish nerve fibers, the threshold would be the best factor and the highest accuracy could be achieved (94.87%) when the cutoff point was set as 0.435 mA. This cutoff point was the safest as it could reach a sensitivity of 100%. However, the specificity was 92.10%. Specifically, 30 dorsal roots possessed a threshold lower than 0.435 mA, among which eight of them were cut 50% according to the rhizotomy protocol. As a matter of fact, 0.435 mA might be adopted as an indicator to prevent the mis-transection of ventral spinal nerves during single-level approach SDR. The result was consistent with the value recommended by Martinez (3). Although one single predictor (threshold) could help surgeons protect ventral roots intraoperatively, the accuracy of identifying nerve roots would not be as high as the value obtained by combining two or three indicators as predictors for the distinguishing of nerve fibers. An accuracy of 95.90% could be acquired by supervised machine learning for the differentiation of ventral/dorsal nerves when threshold + CMAP and threshold + latency were taken as predictors. Currently, the literature describing detailed electrophysiological differences between ventral and dorsal nerve roots of the cauda equina is limited. In addition to the differences reported by Martinez, we were only able to find another two articles. Dai demonstrated that the thresholds of stimulation on ventral roots were 0.3 ± 0.07 mA from L4 to S1, compared with thresholds of dorsal roots with a mean of 2.3 mA (22). De Vloo stated that the threshold of the dorsal root (rootlets) is usually greater than 0.5 mA and not less than 0.2 mA, which is also consistent with our findings (23). When further comparing the thresholds between low-threshold dorsal roots and ventral roots at the same level and on the same side, we found that there was no dorsal root with a threshold lower than that of the paired ventral root at the same level and same side. This indicates that when it is hard to differentiate ventral/dorsal nerve roots (rootlets) during SDR, comparing the roots on the same side and at the same level might be a feasible way.

Finally, an in-depth investigation concerning whether the usage of muscle relaxants would affect the efficiency in distinguishing ventral/dorsal fibers was carried out. As shown in Figure 5, the identification accuracy of nerve roots was high if the stimulation start 40 min after the usage of muscle relaxant. Therefore, it was reasonable to summarize that the trigger stimulation of spinal nerves started at least 40 min after the muscle relaxant. In the medical center where the author worked, rocuronium was used for intubation during the anesthesia in SDR, which was reported to take 35 min to recover 75% from rocuronium, offering strong support for the result acquired in this paper (24, 25).

The study is also subjected to several limitations. Firstly, this is a single-center retrospective study, where relative evidence remains far from sufficient. What's more, the upper limit of stimulation in clinical practice would cause the skew distribution of data. Nonetheless, the median value of the threshold would not be affected. Secondly, the quantitative neurophysiological quantitative data was acquired by triggering the electrical stimulation of nerve fibers. Although single-pulse stimulation is found to possess better stability and repeatability in clinical practice, background noise and manipulation of dissecting spinal nerve roots would still affect EMG amplitudes in monitored muscles. In addition, a few L2 dorsal nerve roots could only elicit muscle contraction in the psoas–iliacus when given stimulation, while the psoas–iliacus was not monitored during the procedure but only clinically observed. Nonetheless, the systematic error and other influences could be corrected by expanding the sample size. As a matter of fact, the results of this study are rather reliable to a certain extent. Thirdly, limited by the single-level approach, characteristics of nerve fibers in other segments were not fully discussed in the study, where only nerves at the L2 segment were included. Perhaps, more detailed data on nerve roots could be obtained in medical centers adopting a multi-level approach SDR.

## Conclusion

Stimulation of ventral and dorsal nerve roots at the L2 segment generates muscle activities in the ipsilateral quadriceps and adductors in most cases. Electro-neurophysiological parameters can guide the classification of ventral/dorsal fibers. Dorsal roots possess a higher threshold than ventral ones, and *Muscle-200* innervated by dorsal roots presents a longer latency and smaller CMAP than those of the ventral ones. Supervised machine learning can efficiently distinguish ventral/dorsal roots with threshold + latency or threshold + CMAP as predictors. Electrical stimulation of nerve fibers is suggested to commence 40 min after applying muscle relaxants.

## Data Availability

The raw data supporting the conclusions of this article will be made available by the authors, without undue reservation.
